# Parallel Mechanism of Spectral Feature-Enhanced Maps in EEG-Based Cognitive Workload Classification

**DOI:** 10.3390/s19040808

**Published:** 2019-02-16

**Authors:** Yihong Zhang, Yuou Shen

**Affiliations:** College of Information Science and Technology, Engineering Research Center of Digitized Textile & Fashion Technology, Ministry of Education, DongHua University, Shanghai 201620, China

**Keywords:** cognitive workload, electroencephalography, spectral map, feature enhancement, neural network, mental workload

## Abstract

Electroencephalography (EEG) provides a non-invasive, portable and low-cost way to convert neural signals into electrical signals. Using EEG to monitor people’s cognitive workload means a lot, especially for tasks demanding high attention. Before deep neural networks became a research hotspot, the use of spectrum information and the common spatial pattern algorithm (CSP) was the most popular method to classify EEG-based cognitive workloads. Recently, spectral maps have been combined with deep neural networks to achieve a final accuracy of 91.1% across four levels of cognitive workload. In this study, a parallel mechanism of spectral feature-enhanced maps is proposed which enhances the expression of structural information that may be compressed by inter- and intra-subject differences. A public dataset and milestone neural networks, such as AlexNet, VGGNet, ResNet, DenseNet are used to measure the effectiveness of this approach. As a result, the classification accuracy is improved from 91.10% to 93.71%.

## 1. Introduction

Electroencephalography (EEG) data is composed of multi-channel bioelectric signals which are recorded by several electrodes and denote changes in bioelectric neural activity [1]. It is noninvasive and low cost and therefore used extensively in research. In the field of human psychology, EEG data is used to identify a persons’ emotions, e.g., happiness, sadness, anger and shyness [2]. EEG data is also used in body control research to find changes in body movement (hands/feet-moving, blinking, etc.) and the corresponding brain activity [3,4,5,6]. This paper focuses on people’s cognitive workload since physiological mental fatigue is a significant issue that impacts health and lifestyle [7,8]. In working environments, work stress can exceed the tolerance of people resulting in a decline in working performance. Having control of people’s cognitive workload is important, particularly in activities which require a high level of concentration and attention. For example, engineers in a chemical factory must operate with high precision, and drivers need to maintain a healthy cognitive workload to prevent accidents [9]. Furthermore, workload estimations can be useful for many other applications, e.g., as quantitative measures for ergonometric and usability evaluations, or in medical applications [10].

Of the classical feature extraction methods, common space mode (CSP)-based methods are widely used to identify features [11,12,13], such as common sparse spectral pattern (CSSP) [14], sub-band common spatial pattern (SBCSP) [15], filter bank CSP (FBCSP) [16], discriminative CSP (DCSP) [17], and shrinkage regularized filter bank common spatial patterns (SR-FBCSP) [18] for binary EEG classification. Strong uncorrelated transform complex common spatial patterns (SUTCCSP) have been used to identify different responses of the mu and beta rhythms of EEG traces corresponding to motor imagery tasks [19]. In addition to CSP-based methods, independent component analysis (ICA) is used to filter EEG data [20]. Pinheiro used C4.5 algorithms to control a virtual simulator using EEG signals [21]. In the workload classification area, Wang et al. used wireless EEG Signals to assess memory workload in the n-back task and achieved a general classification accuracy of over 80% [10]. Putze et al. trained and evaluated a Support Vector Machine (SVM)-based workload classifier and achieved a multimodal recognition rate of 95% for the two-class visual task which drops to 73% for cognitive tasks. For the full three-class problem, Putze et al. obtained recognition rates of 70% for the visual and 43% for the cognitive task [22]. Dominic et al. designed a SVM-based system for live recognition of mental workload and achieved an accuracy of 90.7 in the person-dependent evaluation while the accuracy dropped to 72.2 in the person independent evaluation [23]. Recently, Bashivan used Fast Fourier Transformation (FFT) to convert EEG data into the frequency domain and map the 3D spatial positions of electrodes to 2D, according to the distribution of the electrodes. The 4–30 Hz frequency domain containing prime cortical activity is divided into theta (4–7Hz), alpha (8–13Hz) and beta (13–30Hz) frequency bands, generating 3-channel spectral maps which are sent to deep neural networks. This method combines EEG data with deep neural networks and achieve an accuracy of 91.1% [24]. However, the data of the test and training set originated from one cohort of 13 subjects, and therefore if the model was to be used to classify their cognitive workload level of a new volunteer, the accuracy could decrease. Furthermore, FFT was performed on the time series for each trial to estimate the power spectrum of the signal. The sum of squared absolute values within θ, α and β frequency bands was computed and used as separate measurements for each electrode, which causes different spectral ranges of spectral maps from different subjects different trails (the times subject finishing experimental tasks calls trails) [24]. This method extracts information from the three frequency bands and this significantly reduces the quantity of data, however it also compresses the structure information of each trail. 

This study proposes a novel approach to enhance the features of spectral maps and apply some milestone achievements in deep learning to the EEG-based workload classification area. In the feature enhancement part, 3-channel feature-enhanced maps are obtained and combined with original 3-channel spectral maps, thus building the parallel mechanism of spectral feature-enhanced maps which have 6-channels. In the feature extraction part, the shortcut connection in ResNet and short paths in DenseNet improve the ability of feature extraction from the spectral map, and are undertaken for the first time in the EEG-based cognitive workload classification area [25,26]. The final accuracy of cognitive workload classification using a public dataset is improved from 91.1% to 93.6% and the generalization of the network is also improved.

## 2. EEG Dataset and Physiological Underpinnings

In order to compare the effects of the parallel mechanism of the spectral feature-enhanced (PMSFE) maps with spectral maps, the public database provided by Bashivan on GitHub was used in our experiments (Web: https://github.com /pbashivan/ EEGLearn). The dataset records 2670 trails of cognitive workload records from 13 subjects whose age ranged from 24 to 33, obtained with 64 electrodes placed over the scalp at standard 10-10 locations. The method of testing subjects’ cognitive workload was similar to Sternberg’s memory experiment [27,28]. At the beginning, the participants see a number of English letters that are circularly distributed at the same angle in the center of the screen. The letters on the screen are continuously displayed for 0.5 s and participants need to remember these words in 0.5 s. After the letter disappears, participants need to judge whether a random letter appeared after three seconds’ margin. If participants judge correctly, it will be recorded as a valid trail. A total of 2670 trails are recorded in the database and divided into four load levels according to the number of memory letters 2, 4, 6 and 8, respectively. The higher the number of memories, the higher the corresponding load level. 

**Figure 1:**
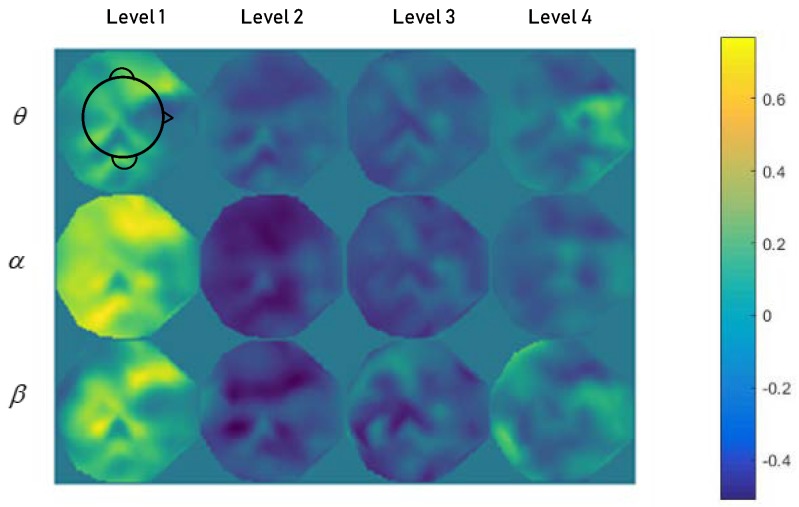
The mean topological spectral maps of *θ*, *α* and *β* at a 4 classes load level. Maps in the first line show the spectral changes in the *θ* frequency band under different load level. Maps in the second line show the spectral changes in the *α* frequency band under different load levels. Maps in the third line show the spectral changes in the *β* frequency band under different load levels. All values have been subtracted from the average spectral value of the whole dataset.

Physiological studies show that prefrontal cortex is the mostly involved during cognitive and memory tasks [29], which is consistent with the features shown in Figure 1, which illustrates the mean topological spectral maps calculated for the *θ*, *α* and *β* frequency bands of different load levels. The color in that prefrontal cortex in level 1 bias blue in all the *θ*, *α* and *β* frequency bands. As the difficulty of memory tasks increases, this area is getting brighter and brighter. What’s more, higher load levels enhance the power in higher frequency bands [30]. The spectral maps at level 2, level 3 and level 4 generally conform to physiological theory except level 1. This is because participants can manage simple memory tasks without paying too much attention. They may didn’t plunge themselves in this task and many ideas which can be considered as noise in daily life may by going on in their mind. At level 2 and higher levels, they have to focus on the task or when they can’t manage the task, then they reduce unnecessary thoughts and the spectral map is helped according to physiological theory.

## 3. Parallel Mechanism of Spectral Feature-Enhanced Maps

### 3.1. The Structure of Parallel Mechanism

**Figure 2:**
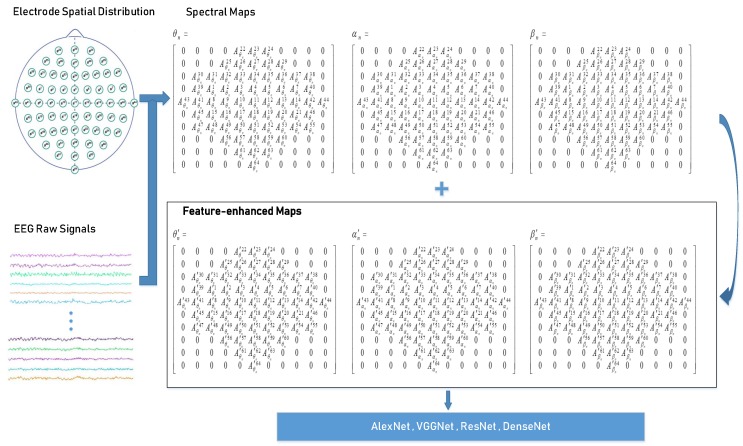
An overview of the approach. (*θ_n_*, *α_n_* and *β_n_* represent the spectral maps at 4–7 Hz, 8–13 Hz, and 13–30 Hz of the No. *n* trail; $A_{\theta_{n}}^{1}\ldots A_{\theta_{n}}^{64},\;A_{\alpha_{n}}^{1}\ldots A_{\alpha_{n}}^{64},\;A_{\beta_{n}}^{1}\ldots A_{\beta_{n}}^{64}$ represent the spectral values recorded by the No. 1 channel electrode to No. 64 channel electrode at 4–7 Hz, 8–13 Hz, and 13–30 Hz of the No. *n* trail).

Figure 2 shows the overview of our approach. EEG raw data from different channels is converted into the frequency domain using FFT and combined with the electrode space information, producing *θ*, *α* and *β* spectral maps [24]. After performing the feature enhancement process, the overall structure features are determined as *θ’*, *α’* and *β’* spectral-enhanced maps.

*θ*, *θ’*, *α*, *α’, β* and *β’* form 6-channel spectral maps, which is the establishment of a parallel mechanism. Finally, PMSFE maps are sent to several milestone neural networks, e.g., AlexNet, VGGNet, ResNet, DenseNet to test for accuracy and generalization [25,26,31,32,33,34].

### 3.2. The Development of PMSFE Maps

The first step is to get the spectral values and form corresponding vectors [24,29]. The 1D vectors of spectral values from the *θ*, *α* and *β* frequency bands are shown as follows:

(1) $$\theta_{n}={\left[A_{\theta_{n}}^{1},A_{\theta_{n}}^{2},A_{\theta_{n}}^{3},A_{\theta_{n}}^{i}\cdots ,A_{\theta_{n}}^{64}\right]}^{T}$$

(2) $$\alpha_{n}={\left[A_{\alpha_{n}}^{1},A_{\alpha_{n}}^{2},A_{\alpha_{n}}^{3},A_{\alpha_{n}}^{i}\cdots ,A_{\alpha_{n}}^{64}\right]}^{T}$$

(3) $$\beta_{n}={\left[A_{\beta_{n}}^{1},A_{\beta_{n}}^{2},A_{\beta_{n}}^{3},A_{\beta_{n}}^{i}\cdots ,A_{\beta_{n}}^{64}\right]}^{T}$$

$A_{\theta_{n}}^{i}$, $A_{\alpha_{n}}^{i}$, $A_{\beta_{n}}^{i}$ represent the spectral values recorded by the No. *i* channel electrode at 4–7 Hz, 8–13 Hz and 13–30 Hz of the No. *n* trail. After considering the electrode spatial distribution, 2D vectors are produced:

(4) $$\begin{array}{c} \theta_{n}/\alpha_{n}/\beta_{n}=\\ \left[\begin{array}{ccccccccccc} 0 & 0 & 0 & 0 & A_{\theta_{n}/\alpha_{n}/\beta_{n}}^{22} & A_{\theta_{n}/\alpha_{n}/\beta_{n}}^{23} & A_{\theta_{n}/\alpha_{n}/\beta_{n}}^{24} & 0 & 0 & 0 & 0\\ 0 & 0 & 0 & A_{\theta_{n}/\alpha_{n}/\beta_{n}}^{25} & A_{\theta_{n}/\alpha_{n}/\beta_{n}}^{26} & A_{\theta_{n}/\alpha_{n}/\beta_{n}}^{27} & A_{\theta_{n}/\alpha_{n}/\beta_{n}}^{28} & A_{\theta_{n}/\alpha_{n}/\beta_{n}}^{29} & 0 & 0 & 0\\ 0 & A_{\theta_{n}/\alpha_{n}/{\beta_{n}}_{n}}^{30} & A_{\theta_{n}/\alpha_{n}/\beta_{n}}^{31} & A_{\theta_{n}/\alpha_{n}/\beta_{n}}^{32} & A_{\theta_{n}/\alpha_{n}/\beta_{n}}^{33} & A_{\theta_{n}/\alpha_{n}/\beta_{n}}^{34} & A_{\theta_{n}/\alpha_{n}/\beta_{n}}^{35} & A_{\theta_{n}/\alpha_{n}/\beta_{n}}^{36} & A_{\theta_{n}/\alpha_{n}/\beta_{n}}^{37} & A_{\theta_{n}/\alpha_{n}/\beta_{n}}^{38} & 0\\ 0 & A_{\theta_{n}/\alpha_{n}/\beta_{n}}^{39} & A_{\theta_{n}/\alpha_{n}/\beta_{n}}^{1} & A_{\theta_{n}/\alpha_{n}/\beta_{n}}^{2} & A_{\theta_{n}/\alpha_{n}/\beta_{n}}^{3} & A_{\theta_{n}/\alpha_{n}/\beta_{n}}^{4} & A_{\theta_{n}/\alpha_{n}/\beta_{n}}^{5} & A_{\theta_{n}/\alpha_{n}/\beta_{n}}^{6} & A_{\theta_{n}/\alpha_{n}/\beta_{n}}^{7} & A_{\theta_{n}/\alpha_{n}/\beta_{n}}^{40} & 0\\ A_{\theta_{n}/\alpha_{n}/\beta_{n}}^{43} & A_{\theta_{n}/\alpha_{n}/\beta_{n}}^{41} & A_{\theta_{n}/\alpha_{n}/\beta_{n}}^{8} & A_{\theta_{n}/\alpha_{n}/\beta_{n}}^{9} & A_{\theta_{n}/\alpha_{n}/\beta_{n}}^{10} & A_{\theta_{n}/\alpha_{n}/\beta_{n}}^{11} & A_{\theta_{n}/\alpha_{n}/\beta_{n}}^{12} & A_{\theta_{n}/\alpha_{n}/\beta_{n}}^{13} & A_{\theta_{n}/\alpha_{n}/\beta_{n}}^{14} & A_{\theta_{n}/\alpha_{n}/\beta_{n}}^{42} & A_{\theta_{n}/\alpha_{n}/\beta_{n}}^{44}\\ 0 & A_{\theta_{n}/\alpha_{n}/\beta_{n}}^{45} & A_{\theta_{n}/\alpha_{n}/\beta_{n}}^{15} & A_{\theta_{n}/\alpha_{n}/\beta_{n}}^{16} & A_{\theta_{n}/\alpha_{n}/\beta_{n}}^{17} & A_{\theta_{n}/\alpha_{n}/\beta_{n}}^{18} & A_{\theta_{n}/\alpha_{n}/\beta_{n}}^{19} & A_{\theta_{n}/\alpha_{n}/\beta_{n}}^{20} & A_{\theta_{n}/\alpha_{n}/\beta_{n}}^{21} & A_{\theta_{n}/\alpha_{n}/\beta_{n}}^{46} & 0\\ 0 & A_{\theta_{n}/\alpha_{n}/\beta_{n}}^{47} & A_{\theta_{n}/\alpha_{n}/\beta_{n}}^{48} & A_{\theta_{n}/\alpha_{n}/\beta_{n}}^{49} & A_{\theta_{n}/\alpha_{n}/\beta_{n}}^{50} & A_{\theta_{n}/\alpha_{n}/\beta_{n}}^{51} & A_{\theta_{n}/\alpha_{n}/\beta_{n}}^{52} & A_{\theta_{n}/\alpha_{n}/\beta_{n}}^{53} & A_{\theta_{n}/\alpha_{n}/\beta_{n}}^{54} & A_{\theta_{n}/\alpha_{n}/\beta_{n}}^{55} & 0\\ 0 & 0 & 0 & A_{\theta_{n}/\alpha_{n}/\beta_{n}}^{56} & A_{\theta_{n}/\alpha_{n}/\beta_{n}}^{57} & A_{\theta_{n}/\alpha_{n}/\beta_{n}}^{58} & A_{\theta_{n}/\alpha_{n}/\beta_{n}}^{59} & A_{\theta_{n}/\alpha_{n}/\beta_{n}}^{60} & 0 & 0 & 0\\ 0 & 0 & 0 & 0 & A_{\theta_{n}/\alpha_{n}/\beta_{n}}^{61} & A_{\theta_{n}/\alpha_{n}/\beta_{n}}^{62} & A_{\theta_{n}/\alpha_{n}/\beta_{n}}^{63} & 0 & 0 & 0 & 0\\ 0 & 0 & 0 & 0 & 0 & A_{\theta_{n}/\alpha_{n}/\beta_{n}}^{64} & 0 & 0 & 0 & 0 & 0 \end{array}\right] \end{array}$$

**Figure 3:**
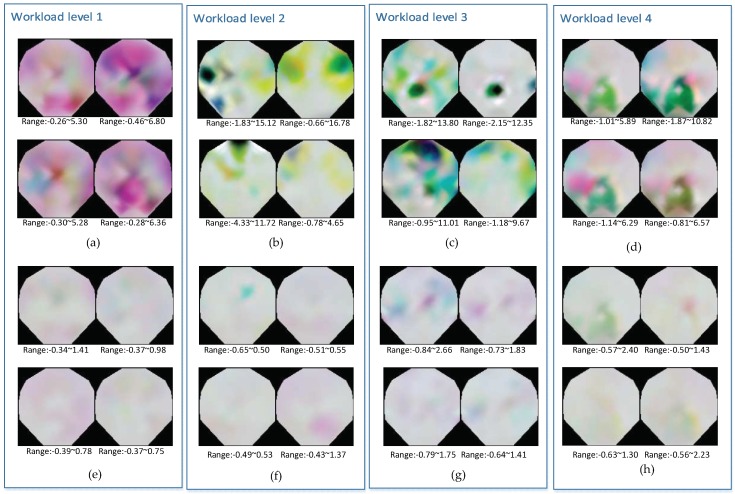
Visualization of examples from four different workload levels. (**a**–**d**) show some samples of maps with obvious features in four classes and the range of spectral value are given. (**e**–**h**) show some samples of maps with unobvious features in four classes and the range of spectral value are given. All these values have been subtracted from the average spectral value of the whole dataset.

These 3-channel maps are delivered into RGB channels and the visualization pictures are shown in Figure 3. Significant features are identified in the upper parts of the four workload levels, while features in the lower parts are not obvious. This could be due to the colorful pictures which indicate that trails have a wider range of maximum and minimum values, while pictures with unremarkable features don’t have a large fluctuation range of spectral power values. In the four cognitive load levels, pictures with unremarkable features are very common, indicating the 2670 trails range should be considered and every trail needs to be enhanced to fully identify the structural information. If these spectral maps are only regulated in the whole standard, the structure information of maps with unremarkable features would be compressed. Since EEG data is very sensitive, any slight body movement or distraction of mind could result in significant changes in the spectral map and influence the final result. Therefore, in addition to the power information of each electrode frequency domain, the whole structure of the spectral map also needs to be considered. An individual’s cognitive workload level is related to its spectral power and also the overall spatial distribution. Spectral maps that have not been enhanced on a self-based basis can weaken the expression of the structure information in deep neural networks.

The details of enhancing the structure features of spectral map are described as follows. After the *θ_n_*, *α_n_* and *β_n_* spectral power values have been subtracted from the overall average, Equation (5) is used to project all the *θ_n_*, *α_n_* and *β_n_* into a range from 0 to 1, and $\theta_{n}^{*},\;\alpha_{n}^{*}$ and $\beta_{n}^{*}$ are thus obtained:

(5) $$A_{n}^{i*}=\frac{1}{1+e^{-0.15\cdot A_{n}^{i}}}$$

Since the spectral power is calculated by the sum of squared absolute values within each of the *θ_n_*, *α_n_* and *β_n_* frequency bands, Equation (5) could weaken the impact of the square operation and avoid the interference from abnormal large values. Equation (8) is used to widen the range of minimum and maximum values:

(6) $$Max(A_{n}^{i*})=Max(A_{\theta_{n}}^{1*},A_{\theta_{n}}^{2*}\cdots ,A_{\theta_{n}}^{i*}\cdots ,A_{\theta_{n}}^{64*},A_{\alpha_{n}}^{1*},A_{\alpha_{n}}^{2*}\cdots ,A_{\alpha_{n}}^{i*}\cdots ,A_{\alpha_{n}}^{64*},A_{\beta_{n}}^{1*},A_{\beta_{n}}^{2*}\cdots ,A_{\beta_{n}}^{i*}\cdots ,A_{\beta_{n}}^{64*})$$

(7) $$Min(A_{n}^{i*})=Min(A_{\theta_{n}}^{1*},A_{\theta_{n}}^{2*}\cdots ,A_{\theta_{n}}^{i*}\cdots ,A_{\theta_{n}}^{64*},A_{\alpha_{n}}^{1*},A_{\alpha_{n}}^{2*}\cdots ,A_{\alpha_{n}}^{i*}\cdots ,A_{\alpha_{n}}^{64*},A_{\beta_{n}}^{1*},A_{\beta_{n}}^{2*}\cdots ,A_{\beta_{n}}^{i*}\cdots ,A_{\beta_{n}}^{64*})$$

(8) $${A^{\prime }}_{n}^{i}=\frac{A_{n}^{i*}-Min(A_{n}^{i*})}{Max(A_{n}^{i*})-Min(A_{n}^{i*})}$$

$Max\left(A_{n}^{i*}\right)$ is the maximum value of $\theta_{n}^{*},\;\alpha_{n}^{*}$ and $\beta_{n}^{*\;}$ in the No. n trail; $Min\left(A_{n}^{i*}\right)$ is minimum value of $\theta_{n}^{*},\;\alpha_{n}^{*}$ and $\beta_{n}^{*\;}$ the in No. n trail.

Substituting Equation (5) into Equation (8), the final feature-enhanced equation and spectral feature-enhanced maps are obtained as follows:

(9) $${A^{\prime }}_{n}^{i}=\frac{(e^{Min(-0.15A_{n}^{i})}-e^{-0.15A_{n}^{i}})\cdot \left(1+e^{Max(-0.15A_{n}^{i})}\right)}{(e^{Min(-0.15A_{n}^{i})}-e^{Max(-0.15A_{n}^{i})})\cdot \left(1+e^{-0.15A_{n}^{i}}\right)}$$

(10) $$\begin{array}{c} \theta_{n}^{\prime }/\alpha_{n}^{\prime }/\beta_{n}^{\prime }=\\ \left[\begin{array}{ccccccccccc} 0 & 0 & 0 & 0 & A_{\theta_{n}/\alpha_{n}/\beta_{n}}^{\prime 22} & A_{\theta_{n}/\alpha_{n}/\beta_{n}}^{\prime 23} & A_{\theta_{n}/\alpha_{n}/\beta_{n}}^{\prime 24} & 0 & 0 & 0 & 0\\ 0 & 0 & 0 & A_{\theta_{n}/\alpha_{n}/\beta_{n}}^{\prime 25} & A_{\theta_{n}/\alpha_{n}/\beta_{n}}^{\prime 26} & A_{\theta_{n}/\alpha_{n}/\beta_{n}}^{\prime 27} & A_{\theta_{n}/\alpha_{n}/\beta_{n}}^{\prime 28} & A_{\theta_{n}/\alpha_{n}/\beta_{n}}^{\prime 29} & 0 & 0 & 0\\ 0 & A_{\theta_{n}/\alpha_{n}/{\beta_{n}}_{n}}^{\prime 30} & A_{\theta_{n}/\alpha_{n}/\beta_{n}}^{\prime 31} & A_{\theta_{n}/\alpha_{n}/\beta_{n}}^{\prime 32} & A_{\theta_{n}/\alpha_{n}/\beta_{n}}^{\prime 33} & A_{\theta_{n}/\alpha_{n}/\beta_{n}}^{\prime 34} & A_{\theta_{n}/\alpha_{n}/\beta_{n}}^{\prime 35} & A_{\theta_{n}/\alpha_{n}/\beta_{n}}^{\prime 36} & A_{\theta_{n}/\alpha_{n}/\beta_{n}}^{\prime 37} & A_{\theta_{n}/\alpha_{n}/\beta_{n}}^{\prime 38} & 0\\ 0 & A_{\theta_{n}/\alpha_{n}/\beta_{n}}^{\prime 39} & A_{\theta_{n}/\alpha_{n}/\beta_{n}}^{\prime 1} & A_{\theta_{n}/\alpha_{n}/\beta_{n}}^{\prime 2} & A_{\theta_{n}/\alpha_{n}/\beta_{n}}^{\prime 3} & A_{\theta_{n}/\alpha_{n}/\beta_{n}}^{\prime 4} & A_{\theta_{n}/\alpha_{n}/\beta_{n}}^{\prime 5} & A_{\theta_{n}/\alpha_{n}/\beta_{n}}^{\prime 6} & A_{\theta_{n}/\alpha_{n}/\beta_{n}}^{\prime 7} & A_{\theta_{n}/\alpha_{n}/\beta_{n}}^{\prime 40} & 0\\ A_{\theta_{n}/\alpha_{n}/\beta_{n}}^{\prime 43} & A_{\theta_{n}/\alpha_{n}/\beta_{n}}^{\prime 41} & A_{\theta_{n}/\alpha_{n}/\beta_{n}}^{\prime 8} & A_{\theta_{n}/\alpha_{n}/\beta_{n}}^{\prime 9} & A_{\theta_{n}/\alpha_{n}/\beta_{n}}^{\prime 10} & A_{\theta_{n}/\alpha_{n}/\beta_{n}}^{\prime 11} & A_{\theta_{n}/\alpha_{n}/\beta_{n}}^{\prime 12} & A_{\theta_{n}/\alpha_{n}/\beta_{n}}^{\prime 13} & A_{\theta_{n}/\alpha_{n}/\beta_{n}}^{\prime 14} & A_{\theta_{n}/\alpha_{n}/\beta_{n}}^{\prime 42} & A_{\theta_{n}/\alpha_{n}/\beta_{n}}^{\prime 44}\\ 0 & A_{\theta_{n}/\alpha_{n}/\beta_{n}}^{\prime 45} & A_{\theta_{n}/\alpha_{n}/\beta_{n}}^{\prime 15} & A_{\theta_{n}/\alpha_{n}/\beta_{n}}^{\prime 16} & A_{\theta_{n}/\alpha_{n}/\beta_{n}}^{\prime 17} & A_{\theta_{n}/\alpha_{n}/\beta_{n}}^{\prime 18} & A_{\theta_{n}/\alpha_{n}/\beta_{n}}^{\prime 19} & A_{\theta_{n}/\alpha_{n}/\beta_{n}}^{\prime 20} & A_{\theta_{n}/\alpha_{n}/\beta_{n}}^{\prime 21} & A_{\theta_{n}/\alpha_{n}/\beta_{n}}^{\prime 46} & 0\\ 0 & A_{\theta_{n}/\alpha_{n}/\beta_{n}}^{\prime 47} & A_{\theta_{n}/\alpha_{n}/\beta_{n}}^{\prime 48} & A_{\theta_{n}/\alpha_{n}/\beta_{n}}^{\prime 49} & A_{\theta_{n}/\alpha_{n}/\beta_{n}}^{\prime 50} & A_{\theta_{n}/\alpha_{n}/\beta_{n}}^{\prime 51} & A_{\theta_{n}/\alpha_{n}/\beta_{n}}^{\prime 52} & A_{\theta_{n}/\alpha_{n}/\beta_{n}}^{\prime 53} & A_{\theta_{n}/\alpha_{n}/\beta_{n}}^{\prime 54} & A_{\theta_{n}/\alpha_{n}/\beta_{n}}^{\prime 55} & 0\\ 0 & 0 & 0 & A_{\theta_{n}/\alpha_{n}/\beta_{n}}^{\prime 56} & A_{\theta_{n}/\alpha_{n}/\beta_{n}}^{\prime 57} & A_{\theta_{n}/\alpha_{n}/\beta_{n}}^{\prime 58} & A_{\theta_{n}/\alpha_{n}/\beta_{n}}^{\prime 59} & A_{\theta_{n}/\alpha_{n}/\beta_{n}}^{\prime 60} & 0 & 0 & 0\\ 0 & 0 & 0 & 0 & A_{\theta_{n}/\alpha_{n}/\beta_{n}}^{\prime 61} & A_{\theta_{n}/\alpha_{n}/\beta_{n}}^{\prime 62} & A_{\theta_{n}/\alpha_{n}/\beta_{n}}^{\prime 63} & 0 & 0 & 0 & 0\\ 0 & 0 & 0 & 0 & 0 & A_{\theta_{n}/\alpha_{n}/\beta_{n}}^{\prime 64} & 0 & 0 & 0 & 0 & 0 \end{array}\right] \end{array}$$

**Figure 4:**
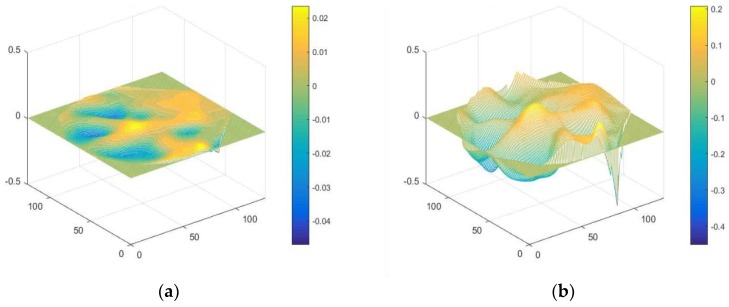

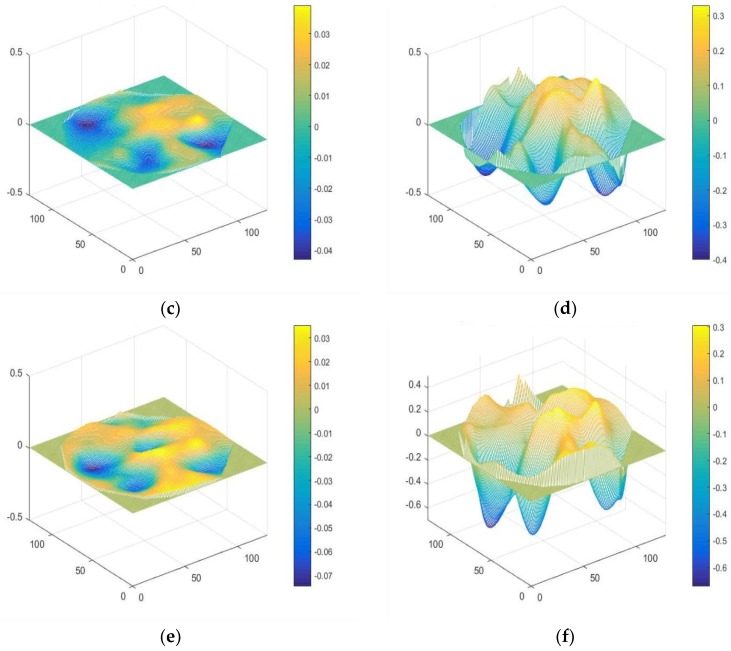
(**a**) 3D image of the *θ* channel; (**b**) 3D image of the feature-enhanced *θ*’ channel; (**c**) 3D image of the *α* channel; (**d**) 3D image of the feature-enhanced *α’* channel; (**e**) 3D image of the *β* channel; (**f**) 3D image of the feature-enhanced *β’* channel.

The original 3-channel spectral maps *θ_n_*, *α_n_* and *β_n_* and the feature-enhanced 3-channel maps $\theta_{n}^{\prime },\;\alpha_{n}^{\prime }$ and $\beta_{n}^{\prime \;}$ form the 6-channel maps, which build the PMSFE maps since the sum of squared absolute values within each of the three frequency bands was computed and used as separate measurements for each electrode [24]. This way of calculation can extract main information effectively and realize real-time processing but may cause different ranges of spectral value. Equation (9) can reduce the interference from extreme values and improve the expression of compressed structural information. Figure 4 contrasts the 3D images of the *θ*, *α* and *β* channels and the enhanced *θ’*, *α’* and *β’* channels and the explanation of mentioned notations are given in Table 1.

## 4. Baseline Methods and Training Process

To measure the effect of PMSFE maps compared to unenhanced spectral maps, most popular and milestone deep neural networks, such as AlexNet, VGGNet, ResNet, and DenseNet are used as baseline methods [25,26,31,32,33,34]. In the feature-extracting layers, *θ*, *α*, *β* and *θ’*, *α’*, *β’* are trained separately and do not affect each other. In the classification layer, they are fully linked with each other.

**Figure 5:**
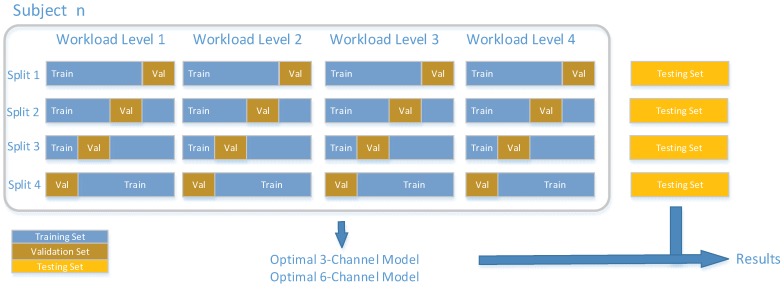
The process of stratified 4-fold cross validation and final testing. Training set and validation set accounts for 80% which is used to find the optimal 3-channel model and 6-channel model. Then the remaining 20% testing set was used to calculate the final accuracy.

The proportion of training set, validation set and test set is 6:2:2 which is randomly divided according to every workload level of every subject proportionately. Stratified cross validation is used to find the optimal model [35]. Part (80%) of the whole data set is divided into four folds. We take turns to use each fold as the validation set and use the remaining three folds as the training set (Figure 5). Every split can get the error of the model and the average error of the four errors indicates the model performance. By adjusting the hyperparameters, an optimal 3-channel model and 6-channel model can be found. The matrix of the optimal model is saved and transferred when using the testing set. The training set is trained based on Pytorch on an Nvidia GTX-1080TI GPU. During the process of training, transfer learning with a learning rate of 0.001 and momentum 0.9 is used to reduce the training time [36,37,38].

Some basic information and parameter settings are introduced and the corresponding validation loss line during training are given correspondingly. The convergence speed and state of neural networks can be directly observed through the change of validation loss.

### 4.1. AlexNet

**Figure 6:**
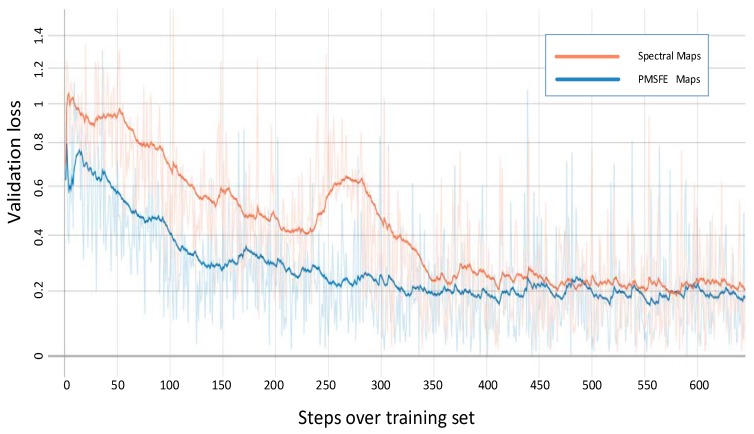
Validation loss of spectral maps and PMSFE maps over training set using AlexNet. (Smooth = 0.95 in Tensorboardx).

Due to limited GPU computing capacity in its day, AlexNet was divided into two parts. Today, improved GPU computing power has solved the problem and some slight changes have been made. In the feature learning section, Bashivan’s method is used to form the topology-preserving spectral maps in 224 × 224 size [24]. The three input channels are extended into six channels, the size of kernel in five convolutional layers is 11 × 11, 5 × 5, 3 × 3, 3 × 3 and 3 × 3 which means the sizes of the matrices used to convolve with maps and the corresponding numbers are 64, 192, 384, 256, 256. Usually more kernels exist in latter layers to ensure features of formal layer being fully abstracted. In the classification section, the input and output parameters of the last linear are changed to 4096 and 4 (4096 are the output number of formal layer and 4 is determined by the number of classes). Figure 6 shows the validation loss of spectral maps and PMSFE maps over training set using AlexNet. 

### 4.2. VGGNet

**Figure 7:**
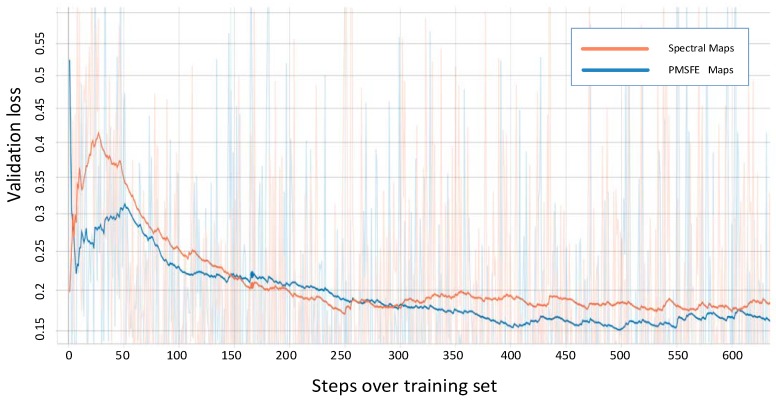
Validation loss of spectral maps and PMSFE maps over the training set in VGGNet-19 (Smooth = 0.97 in Tensorboardx).

On the basis of AlexNet, VGGNet deepened the neural network and the convolutional layers using the same size convolutional filter 3 × 3, achieving greater nonlinearity with less parameters. The vision of VGGNet displayed in the Figure 7 is VGGNet-19. In the feature learning section, the number of kernels in 13 convolutional layers is 64, 64, 128, 128, 256, 256, 256, 512, 512, 512, 512, 512 and 512. The change of classification section is the same in AlexNet.

### 4.3. ResNet

**Figure 8:**
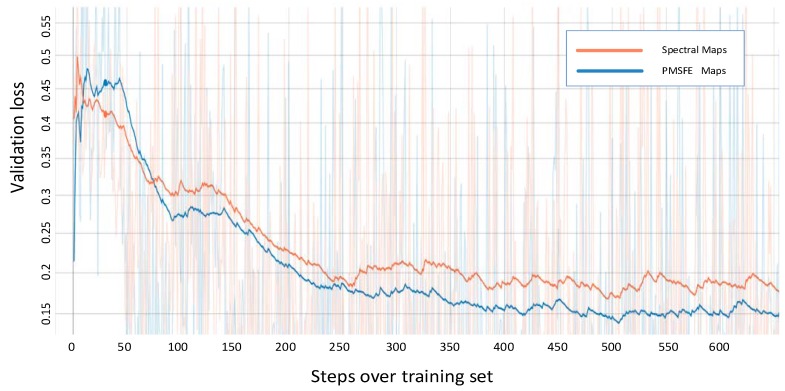
Validation loss of spectral maps and PMSFE maps over training set in ResNet-152. (Smooth = 0.987 in Tensorboardx).

Before the ResNet unit appeared, gradient disappearance and gradient explosions could limit traditional deep networks from being well-trained. In its structure, each block of ResNet consisted of a series of layers and a shortcut connection which connect the input and output of the module and perform the addition operation, addressing gradient disappearance. The validation loss in ResnetV2-152 is recorded as an example to demonstrate the difference between spectral maps and PMSFE maps in Figure 8 and the output of its fully connected layers is also changed to 4.

### 4.4. DenseNet

**Figure 9:**
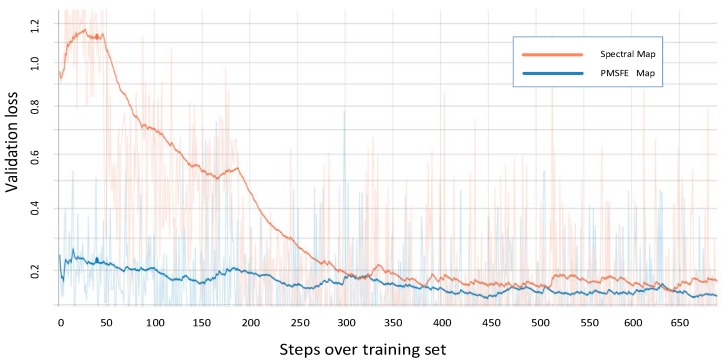
Validation loss of spectral maps and PMSFE maps over training set in DenseNet-201. (Smooth = 0.978 in Tensorboardx).

Unlike ResNet, which adds the output to the input to form a residual structure, DenseNet parallels the output to the input, enabling each layer to directly get the output of all the previous layers. Each layer of DenseNet only requires a few learnt features, significantly reducing the parameters and computation required. The validation loss in Densenet-201 is recorded as an example to demonstrate the difference between spectral maps and PMSFE maps in Figure 9 and the output of its fully connected layers is also changed to 4.

The two colored lines represent the validation loss of PMSFE maps and spectral maps in the four kinds of network and the cross-entropy loss function is used to calculate the loss value.

All four validation loss lines demonstrate that PMSFE maps have a lower validation loss compared to spectral maps and the difference ranges from 0.010 to 0.022, indicating that the parallel mechanism creates a smoother training process and eventually stabilizes at a lower level.

## 5. Results and Discussions

The effect of a parallel mechanism is tested from two aspects. In the first aspect, the accuracy of Spectral Maps and PMSFE maps are calculated according to four trained models: AlexNet, VGGNet, ResNet and DenseNet. The second aspect is the testing of generalizations between spectral and PMSFE maps in neural networks.

### 5.1. Accuracy

Table 2 shows the final accuracy of spectral maps and PMSFE maps in nine models, and the increased rates after using a parallel mechanism. By adding feature-enhanced maps into the network the accuracy of AlexNet and VGGNet are increased from 88.77% to 90.18%, from 91.46% to 92.77% in 16-layers vision with batch normalization (BN) and from 91.70% to 93.34% in 19-layers vision with BN [39]. Although the mean accuracy of VGGNet performs better than AlexNet, the rate of increase is similar (approximately 1.5%).

The rate of increase is approximately 1% in ResNet and 0.56% in DenseNet, due to the feature-enhanced map enhancing the structure information using every trail. This phenomenon also reflects the ability of DenseNet and ResNet to extract features better than AlexNet’s and VGGNet’s.

The highest accuracy is 93.71% using PMSFE maps in ResNet-152, which increases the final accuracy from 91.1% to 93.71%. Across the rate increase of 2.61%, 1.52% is due to performance improvements of the network, and 1.09% is due to the parallel mechanism of spectral feature-enhanced maps.

**Figure 10:**
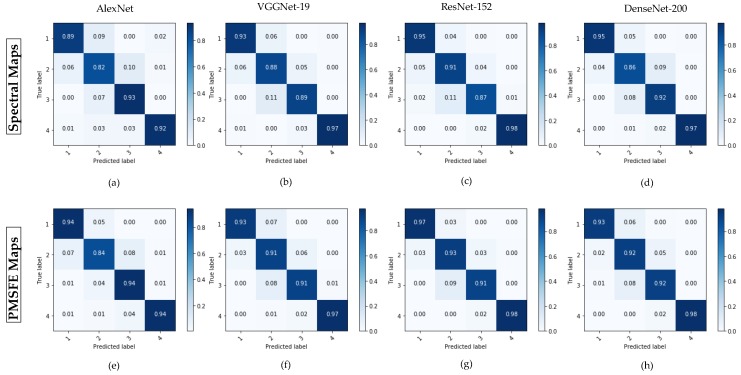
The confusion matrix of spectral maps and PMSFE maps. (**a**) The confusion matrix of spectral maps in AlexNet. (**b**) The confusion matrix of spectral maps in VGGNet-19. (**c**) The confusion matrix of spectral maps in ResNet-152. (**d**) The confusion matrix of spectral maps in DenseNet-200. (**e**) The confusion matrix of PMSFE maps in AlexNet. (**f**) The confusion matrix of PMSFE maps in VGGNet-19. (**g**) The confusion matrix of PMSFE maps in ResNet-152. (**h**) The confusion matrix of PMSFE maps in DenseNet-200.

Figure 10 shows the confusion matrix of spectral maps and PMSFE maps in AlexNet, VGGNet-19, ResNet-152 and DenseNet-200. It obvious that load level 2 and load level 3 are easily confused when using original spectral maps. The probabilities of the neural network misclassifying level 3 into level 2 are correspondingly 7%, 11%,11% and 8%. The percentages decrease to 4%, 8%, 9% and 8% after using PMSFE maps.

### 5.2. Generalization

In actual usage of cognitive workload classification, the final accuracy of new subjects is not necessarily so high. Although the training and test set don’t overlap, they are all from the same 13 subjects, which results in features of specific subjects being already trained by deep neural networks and the accuracy would be higher. In the testing of generalization after using PMSFE maps, trails of specific subjects are identified as the test set, and these trails from the remaining 12 subjects is used as training set. For example, in Table 3 the EEG data from subject No.1 is not included when training the network and is only used as the test set. This method simulates the actual application and guarantees the validity generalization.

ResNet-152 which performs best in final accuracy is used to test the generalization. From Table 3, the effect of a parallel mechanism can be identified clearly. Most subjects have an increase in the range from 0.46% to 2.70%, due to the spectral value of different trails from different subjects having different ranges.

## 6. Conclusions and Challenges

The use of deep neural networks is widespread, while the application of frontier neural networks in EEG-based cognitive workload classification has been delayed for a certain period. One main factor which limits the use of neural networks in EEG-based classification is the lack of experimental data. In this study, we introduce the traditional methods used to dealing with EEG data, the recent works by Bashivan in the workload classification area and use his public dataset recording 2670 trails of experimental data. Compared to other public datasets only recording trails no more than a few hundred, the dataset can meet the training needs of neural networks. In this study, the method of using spectral maps is improved and a parallel mechanism of spectral feature-enhanced maps is proposed addressing the problems with compressed structural information.

The final results demonstrate the effects of adding spectral feature-enhanced maps into a network depends on individuals. In the test of generalization among 13 subjects, the maximum increase ratio is 2.7%. The final accuracy test has a mean value of 93.71% with a standard deviation of 0.0027.

Actually, the monitoring of cognitive load is still in the laboratory stage. This is partly because EEG raw signals are extremely large and when using mobile EEG facilities there is usually a delay. Some breakthroughs in wireless sensor networks, cloud computing and mobile edge computing (MEC) can manage these problems. Aloqaily et al. built a generalization of quality-of-experience (QoE) design in the cloud computing area and eventually fulfilled the service requirements by compromising between delay, service cost and information revealed to the TTP [40]. Baker et al. proposed a GreeDi-based reactive routing protocol aimed at selecting the most efficient route in terms of energy consumption between two nodes in VANETs [41]. Otoum et al. built a hierarchical trust-based WSN monitoring model for the smart grid equipment in order to detect Black-Hole (B-H) attacks and the proposed Adaptively Supervised and Clustered Hybrid IDS (ASCH-IDS) for wirelessly connected sensor performs at 98.9% detection rate and approximately 99.80% overall accuracy to detect known and unknown malicious behavior in a sensor network [42,43]. Meneguette et al. proposed an effective mobile content delivery solution for network delivery and increased content availability without compromising network overhead, regardless of traffic conditions and road networks [44]. Ridhawi et al. introduced a fog-to-fog (F2F) data caching and selection method, which allows IoT devices to retrieve data in a faster and more efficient way. Their presented solution provides guaranteed and fast delivery of the requested cloud composite services to end users while sustaining QoS requirements and load balancing among edge and mobile nodes [45]. As the edge computing paradigm began to take precedence, a mobile device cloud (MDC) formed at the edge based on idle intra-device resources emerged [46,47]. All these novel breakthroughs provide technical support for a wider usage of EEG-based monitoring, which is our future work.

## Figures and Tables

**Table 1:** The explanation of variables.

Notation	Explanation
*θ_n_*	The spectral values in the frequency bands of theta (4–7 Hz)
*α_n_*	The spectral values in the frequency bands of alpha (8–13 Hz)
*β_n_*	The spectral values in the frequency bands of beta (13–30 Hz)
$A_{n}^{i}$/$A_{\theta_{n},\alpha_{n},\beta_{n}}^{i}$	The spectral values recorded by No. *i* channel electrode at 4–7 Hz, 8–13 Hz and 13–30 Hz of the No. *n* trail
$A_{n}^{i*}$/$A_{\theta_{n},\alpha_{n},\beta_{n}}^{i*}$	The spectral value in No. *i* channel electrode at 4–7 Hz, 8–13 Hz and 13–30 Hz of the No. *n* trail processed by Equation (5)
$A_{n}^{\prime i}$/$A_{\theta_{n},\alpha_{n},\beta_{n}}^{\prime i}$	The final enhanced spectral value in No. *i* channel electrode at 4–7 Hz, 8–13 Hz and 13–30 Hz of the No. *n* trail processed by Equation (9)
$\theta_{n}^{*}$	The spectral values in the *θ* (4–7 Hz) frequency bands processed by Equation (5)
$\alpha_{n}^{*}$	The spectral values in the *α* (8–13 Hz) frequency bands processed by Equation (5)
$\beta_{n}^{*}$	The spectral values in the *β* (13–30 Hz) frequency bands processed by Equation (5)
$Max\left(A_{n}^{i*}\right)$	The maximum value of $\theta_{n}^{*}$, $\alpha_{n}^{*}$ and $\beta_{n}^{*}$ in the No. *n* trail
$Min\left(A_{n}^{i*}\right)$	The minimum value of $\theta_{n}^{*}$, $\alpha_{n}^{*}$ and $\beta_{n}^{*}$ in the No. *n* trail
$\theta_{n}^{\prime }$	The enhanced *θ* (4–7 Hz) frequency bands
$\alpha_{n}^{\prime }$	The enhanced *α* (8–13 Hz) frequency bands
$\beta_{n}^{\prime }$	The enhanced *β* (13–30 Hz) frequency bands

**Table 2:** The accuracy of spectral maps and PMSFE maps across all networks and the rate of increase after using PMSFE maps.

Network	Spectral Maps		PMSFE Maps		Increased Rate (%)
	Mean Accuracy (%)	Standard Deviation	Mean Accuracy (%)	Standard Deviation	
AlexNet	88.78	0.0019	90.18	0.0031	1.4
VGGNet-16BN	91.46	0.0026	92.77	0.0030	1.31
VGGNet-19BN	91.70	0.0016	93.34	0.0026	1.64
ResNet-50	92.50	0.0027	93.08	0.0023	0.58
ResNet-101	92.61	0.0025	93.68	0.0032	1.07
ResNet-152	92.62	0.0023	93.71	0.0027	1.09
DenseNet-121	92.33	0.0051	92.87	0.0039	0.54
DenseNet-169	92.56	0.0029	93.08	0.0027	0.52
DenseNet-201	92.88	0.0028	93.47	0.0021	0.59

**Table 3:** The accuracy of spectral maps and PMSFE maps tested on 13 subjects and the rate of increase after using PMSFE maps.

Subject	Spectral Maps (%)	PMSFE Maps (%)	Increased Rate (%)
1	55.68	58.38	2.70
2	70.28	72.17	1.89
3	88.95	91.46	2.51
4	99.50	99.50	0
5	99.49	100	0.51
6	99.00	99.5	0.50
7	99.00	100	1.00
8	100	100	-
9	100	100	-
10	100	100	-
11	98.62	99.08	0.46
12	72.25	72.25	0
13	46.36	47.73	1.37
